# ^68^Ga-DOTATATE PET/CT and MRI with Diffusion-Weighted Imaging (DWI) in Short- and Long-Term Assessment of Tumor Response of Neuroendocrine Liver Metastases (NELM) Following Transarterial Radioembolization (TARE)

**DOI:** 10.3390/cancers13174321

**Published:** 2021-08-27

**Authors:** Maria Ingenerf, Sophia Kiesl, Salma Karim, Leonie Beyer, Harun Ilhan, Johannes Rübenthaler, Max Seidensticker, Jens Ricke, Christine Schmid-Tannwald

**Affiliations:** 1 Klinik und Poliklinik für Radiologie, Klinikum der Universität München, LMU München, Marchioninistr. 15, 81377 Munich, Germany; sophia.kiesl@med.uni-muenchen.de (S.K.); salma.ka@gmx.de (S.K.); johannes.ruebenthaler@med.uni-muenchen.de (J.R.); max.seidensticker@med.uni-muenchen.de (M.S.); Jens.Ricke@med.uni-muenchen.de (J.R.); christine.schmid-tannwald@med.uni-muenchen.de (C.S.-T.); 2 Department of Nuclear Medicine, University Hospital, LMU Munich, Marchioninistr. 15, 81377 Munich, Germany; leonie.beyer@med.uni-muenchen.de (L.B.); Harun.Ilhan@med.uni-muenchen.de (H.I.)

**Keywords:** DWI, response assessment, neuroendocrine tumors, TARE, PET/CT

## Abstract

**Simple Summary:**

TARE with ^90^Yttrium has become a valuable treatment option for patients with unresectable NELMs. However, early evaluation of therapy response remains challenging as size-based response assessments (such as RECIST) are known to be limited, especially in slow-growing tumors. Alternatives such as quantitative evaluation of SUV of ^68^Ga-DOTATATE PET/CT and ADC of DWI-MRI have not been compared so far. We found that early percentage changes in SUV tumor-to-organ ratios on first follow-up after TARE could predict longer HPFS in patients with NELM and were superior to ΔSUVmax/SUVmean alone or to ΔADC.

**Abstract:**

The aim of this study was to evaluate the role of SUV and ADC in assessing early response in patients with NELM following TARE. Thirty-two patients with pre- and postinterventional MRI with DWI and ^68^Ga-DOTATATE PET/CT were included. ADC and SUV of three target lesions and of tumor-free spleen and liver tissue were determined on baseline and first follow-up imaging, and tumor to spleen (T/S) and tumor to liver (T/L) ratios were calculated. Response was assessed by RECIST 1.1 and mRECIST on first follow-up, and long-term response was defined as hepatic progression-free survival (HPFS) over 6, 12, and <24 months. In responders, intralesional ADC values increased and SUV decreased significantly regardless of standard of reference for response assessment (mRECIST/RECIST/HPFS > 6/12/24 m). Using ROC analysis, ΔSUV T/S ratio (max/max) and ΔSUV T/L ratio (max/mean) were found to be the best and most robust metrics to correlate with longer HPFS and were superior to ΔADC. ΔT/S ratio (max/max) < 23% was identified as an optimal cut-off to discriminate patients with longer HPFS (30.2 m vs. 13.4 m; *p* = 0.0002). In conclusion, early percentage changes in SUV tumor-to-organ ratios on first follow-up seem to represent a prognostic marker for longer HPFS and may help in assessing therapeutic strategies.

## 1. Introduction

TARE with ^90^Yttrium has become a valuable treatment option for patients with unresectable NELMs [1], but short-term evaluation of tumor response after TARE remains challenging. The presence of liver metastases is one of the most powerful factors influencing survival, and effective and safe therapy of hepatic metastases can extend survival and improve quality of life. Therefore, short-term evaluation of therapy response following TARE is important in adapting the therapy concept as soon as possible in the event of an insufficient or even faulty response. Conventionally, therapy response in oncological clinical studies is assessed according to RECIST 1.1, which is based on changes in tumor diameter. However, several reports suggest that size-based evaluation alone is not accurate in monitoring response after locoregional treatments [2], which is especially relevant for slow-growing tumors such as differentiated NETs. Therefore, it could be interesting to evaluate if mRECIST (which is based on size changes in enhancing tumor parts) allows/improves therapy monitoring in patients with NELM, although it is primarily recommended for patients with HCC. To address these limitations, other parameters such as a reduction of somatostatin receptor (SSTR)-expressing tumor cells, declining vascularization, or a decreasing cellularity could serve as additional parameters to assess therapy response and might even be considered as prognostic factors. Furthermore, regarding the assessment of the treatment response, not only the short-term follow-up should be evaluated, but also and especially the long-term follow-up in terms of progression-free survival and overall survival.

Good-to-moderately differentiated NETs characteristically overexpress somatostatin receptors (SSR). This property is used in functional imaging with ^68^Ga-labeled somatostatin analogues (SSA) (^68^Ga-DOTA-TATE, -DOTA-NOC, and -DOTA-TOC) with PET/CT, which enables detection of neuroendocrine primary tumors and lymphogenic and hematogenic metastases with high sensitivity and specificity [3]. PET/CT with ^68^Ga-DOTA-TATE/-NOC/-TOC is recommended for initial staging and follow-up of neuroendocrine gastroenteropancreatic tumors by the European Society for Medical Oncology Guidelines Working Group [4]. Unlike ^18^F-FDG PET/CT which is well-established in treatment monitoring of various tumor types, such as malignant lymphoma [5] or non-small-cell lung cancer [6] and has well-defined response criteria such as the Deauville criteria or Positron Emission tomography Criteria in Solid Tumors (PERCIST) [7], quantitative evaluation of SSR imaging to monitor treatment response is not yet standardized. Several studies suggest that these tracers could serve as a prognostic parameter in NET patients undergoing octreotide treatment [8], peptide-receptor radionuclide therapy (PRRT) [9], or TARE [10]. 

In contrast to PET/CT, liver MRI has a higher soft tissue contrast and thus a higher sensitivity and specificity in the detection of liver metastases, especially using liver-specific contrast media as Gd-EOB DTPA. Therefore, if both are available, it is used complementary to PET/CT for staging patients with NET. In addition to purely morphological sequences, diffusion imaging enables statements regarding cellularity which can be quantitatively analyzed in the form of apparent diffusion coefficients (ADC). In oncologic imaging, DWI is increasingly and routinely used for the detection and characterization of liver lesions [11] since malignant lesions usually show lower ADC values than benign lesions [12,13,14]. In addition DWI is increasingly used in therapy assessment; changes in signal intensity of the lesions in DWI as well as corresponding changes in ADC values due to increasing tumor necrosis during therapy can be seen before a change in size or enhancement [15]. In addition, DWI can be used to determine tumor grading and it has been shown that there is an association between ADC values and Ki-67. Besa et al. demonstrated in their study, that the ADC-mean of G3 tumors was significantly lower than that of G1 and G2 tumors [16]. In a large meta-analysis, Surov et al. found a strong correlation between ADC mean and Ki-67 in ovarian cancer, urothelial carcinomas, cerebral lymphoma, and also in neuroendocrine tumors [12]. Furthermore, other histopathological parameters are associated with ADC values. In a recent study it was shown that ADCmean was negatively associated with average nucleic area, and total nucleic area in meningiomas [17]. In other tumors, there were also associations between ADC and cell count such as glioma, ovarian cancer, or lung cancer [18]. Some studies supposed that early increase of ADC values in hepatic metastasis of various primary tumors (including NET) following TARE was associated with better prognosis [19,20]. 

So far, studies analyzing the role of ^68^Ga-DOTA-TATE PET/CT to assess treatment response in patients with NELMs following TARE are largely missing and measurement methods such as standardized uptake values (SUV) and tumor-to-organ uptake ratios (liver or spleen) are utilized heterogeneously. Therefore, the aim of this study was to evaluate both modalities in patients with NELM undergoing TARE with regard to parameters that are most accurate to assess early therapy response. 

## 2. Materials and Methods

### 2.1. Patients

**Figure 1:**
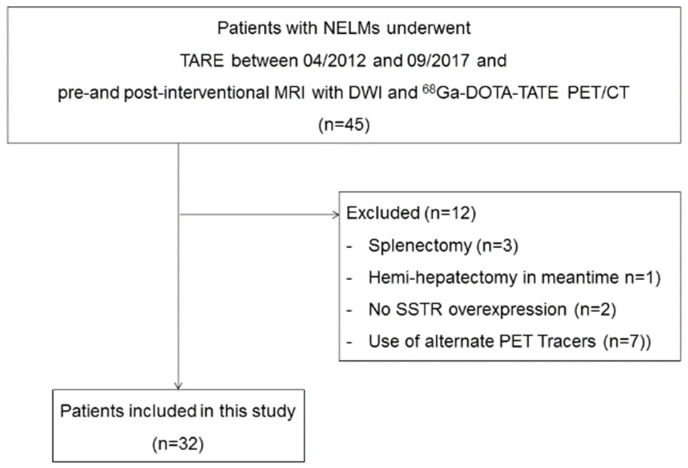
Inclusion and exclusion criteria.

Patients with liver metastases of NET of different primary tumor sites who were treated by TARE with ^90^Y at our department between April 2012 and September 2017 and who each had one MRI with DWI and one ^68^Ga-DOTA-TATE PET/CT before and after therapy were included. Baseline imaging was performed 42 days (±24 days) (MRI) and 81 days (±50 days) (PET-CT) before therapy, and follow-up imaging was acquired 100 d (±50 days) (MRI) and 95 d (±21 days) (PET-CT) after first TARE session, respectively. Exclusion criteria were a splenectomy before TARE or first follow-up scan, a missing SSTR overexpression, a lesion size of less than 1 cm, and severe motion artefacts (Figure 1). The local research ethics committee approved this retrospective study and waived need for written informed patient consent. 

### 2.2. TARE

For all patients in this study the therapy decision for TARE was based on consensus in an multidisciplinary tumor conference. The procedure was performed as described elsewhere [19,21]. Before treatment, suitability of each patient was assessed by performing a hepatic angiography and a technetium-99m-macroaggregated albumin (99mTc-MAA) single photon emission computed tomography/computed tomography (SPECT/CT) scan to calculate liver-to-lung shunt fraction. Aberrant vessels were coil-embolized before the treatment session. Planar and SPECT imaging was performed to simulate possible extrahepatic sphere deposition and pulmonary shunting. The prescribed activity was estimated pursuant to the modified body surface area (BSA) method [21]. During treatment a microcatheter was selectively placed at a previously defined target vessel and a suspension consisting of resin spheres labelled with Yttrium90 (SIR-Spheres^®^; Sirtex Medical Limited, North Sydney, Australia) in sterile water was injected. 

### 2.3. MR Imaging 

MR examinations were performed on a 1.5 T MR system (Magnetom Avanto (*n* = 28); Magnetom Aera (*n* = 36) Siemens Healthcare, Erlangen, Germany; and Ingenia S, Philips Healthcare, Hamburg, Germany) using a phased-array-coil for signal reception. Our usual liver imaging protocol contained unenhanced T1w gradient-echo (GRE) sequences in- and out-of-phase, a single shot T2w sequence, a T1w 3D GRE sequence with fat suppression (fs) before and 20, 50, and 120 s after intravenous contrast injection (Gd-EOB-DTPA; Primovist, Eovist, Bayer Schering Pharma, Germany; 25 µmol/kg body weight), a multishot T2w turbo spin echo sequence (fs), diffusion-weighted sequences with b-values of 50, 400, and 800 s/mm^2^, and a T1w GRE (fs) and a T1w VIBE 3D GRE (fs) after 15 min delay. All sequences were acquired with parallel imaging with an acceleration factor of 2. ADC maps were calculated with all b-values. 

### 2.4. PET/CT

^68^Ga-DOTA-TATE was prepared as previously described [22]. Whole-body PET/CT scans were acquired in three-dimensional mode (3 min per bed position) using a GE Discovery 690 (GE Healthcare, Little Chalfont, UK) (*n* = 3) or a Biograph 64 TruePoint (*n* = 61) PET/CT scanner (Siemens Healthcare, Erlangen, Germany). Imaging was started 60 min after intravenous administration of approximately 200 MBq ^68^Ga-DOTA-TATE, and if possible 20 mg of furosemide. PET/CT scans were performed with a diagnostic CT scan of the neck, thorax, abdomen, and pelvis (100–190 mAs, 120 kV, collimation 2 × 5 mm, pitch of 1.5) and intravenous injection (2.5 mL/s) of an iodine-based contrast agent (Ultravist 300TM; Bayer Healthcare, Berlin, Germany; 1.5 mL/kg body weight) with a delay of 80–110 s in order to depict the portal venous phase of the liver. CT scans were also used for PET attenuation correction. Whole-body PET/CT scans were acquired in three-dimensional mode (3 min per bed position) using a GE Discovery 690 (GE Healthcare, Little Chalfont, UK) or a Biograph 64 TruePoint PET/CT scanner (Siemens Healthcare, Erlangen, Germany). PET images were reconstructed with a transaxial 256 × 256 matrix using VPFX (2 iterations, 36 subsets, 3D Gauss postfilter of 6.5-mm full-width half maximum) for the GE scanner and a transaxial 168 × 168 matrix using TrueX (3 iterations, 21 subsets, 3D Gauss postfilter of 2.0 mm full-width half maximum) for the Biograph scanner. SUV were calculated using the patient’s body weight (SUVbw).

### 2.5. Image Analysis

Pre- and post-interventional MRI images were reviewed by two radiologists (C.S. and M.I., with 14 years and 3 years’ experience in abdominal MRI, respectively), independently, and in two separate sessions. Pre- and postinterventional PET/CTs were reviewed by a third radiologist (L.A.) with experience in nuclear medicine in the same manner. None of the readers were aware of patients’ clinical or follow-up data. Three target lesions were defined for each patient in the treated liver lobe where they appeared best measurable and treatment response according to RECIST and mRECIST was evaluated in consensus. 

For ADC measurements circular regions-of-interest (ROI) were drawn on the slice with the largest extent of the target lesion on DWI-images. Attention was paid to excluding structures close to the rim of the lesion to avoid partial volume effects. These ROIs were transferred to the same slice of the ADCmap to calculate intralesional ADC values including minimal (ADCmin) and mean (ADCmean) ADC (below noted as 10^−3^ mm^2^/s), as these reflect the most commonly assessed ADC values in current literature [23]. In addition, ADC mean values of tumor-free liver were assessed by drawing circular ROIs, as large as possible. Lesion size (recorded as longest diameter (LD)) was measured in the hepatocyte-specific contrast phase on the slice with the largest tumor extent and averaged for each patient for the three target lesions. Baseline and follow-up ADC were averaged between both readers and averaged for each patient for the three target lesions.

^68^Ga-DOTA-TATE uptake was measured as maximum and mean SUV on a dedicated PET workstation (Hermes Medical Solutions, Stockholm Sweden) by semi-quantitatively positioning a circular VOI in the predefined target lesion using a minimum SUV of 4.0. If the tracer uptake was greater than surrounding liver tissue, the lesion was defined as DOTA-TATE-positive. In addition, SUVmax and SUVmean of non-tumorous liver and spleen parenchyma were assessed to calculate tumor-to-organ ratios with tumor-to-spleen (T/S) ratio and tumor-to-liver (T/L) ratio (including SUVmax/SUVmax, SUVmax/SUVmean, and SUVmean/SUVmean). Percentage changes in tumor ADC or SUV at follow-up (T_post_) compared with baseline values (T_pre_) was calculated as:

[(T_post_ − T_pre_)/T_pre_] × 100.

### 2.6. Standard of Reference and Response to Treatment

All patients included in this study had a diagnosis confirmed by histopathology and for most patients Ki-67 labelling index of the primary tumor or liver metastasis and grading according to WHO were obtained. Tumors were classified into three groups by Ki-67 proliferation index according to 2010 WHO Tumor Classification Guideline (G1: Ki-67 Index was <3%, G2: Ki-67 Index was 3–20%, and G3 NET/NEC: Ki-67 Index was >20%) [24]. All patients are treated at one of the European Neuroendocrine Tumor Society Centers of Excellence. 

Treatment response on first follow-up was evaluated according to RECIST 1.1 and mRECIST. Long-term response assessment was evaluated as HPFS over 6, 12, and 24 m, respectively. Hepatic progression-free survival (HPFS) was also calculated from the time of first TARE until progression according to RECIST 1.1. Patients who were still alive at the time of last follow-up (19 January 2021) were censored. OS was determined in days from the first session of TARE until death from any cause or censured at last follow-up.

### 2.7. Statistical Analysis

For statistical analysis Graphpad Prism and SPSS were used (Graphpad Prism Version 6, San Diego, Calif. and SPSS version 25, Chicago, IL, USA) and *p* ≤ 0.05 was regarded as statistically significant. Data distribution was tested for normality by Shapiro–Wilk test and additionally evaluated by visual assessment of the histogram. Variables are given as either mean or median values with standard deviation (SD) or interquartile range (IQR). Pre- and postinterventional ADC values and SUV were compared by Student’s *t*-test. For comparison of percentage changes between different response groups the Mann–Whitney test was used. OS and PFS were analyzed by the Kaplan–Meier curve method, and different groups were compared with log-rank test. Area under the curve (AUC) was calculated from receiver operating characteristics (ROC) analysis and used to determine optimal cut-offs and of ADC and SUV associated with better clinical outcome. Two-way mixed effect intraclass correlation coefficients (ICCs) for absolute agreement across both readers was assessed for ADC measurements. Pearson and Spearman correlation coefficients were calculated for parametric and non-parametric correlation analysis, respectively. 

## 3. Results

### 3.1. Patients’ Cohort and TARE

Thirty-two consecutive patients (16 women, 16 men) with a mean age of 63 ± 10 years met the inclusion criteria with a total of 85 target lesions were included in the analysis. The primary tumor was most commonly localized in the gastrointestinal tract (*n* = 19), and the pancreas (*n* = 7), less common sites were lung (*n* = 3), liver (*n* = 1), and kidney (1). Two NETs were defined as cancers of unknown origin (CUP). With regard to histology, most tumors were categorized as G2 tumors (intermediate grade) (20/32), followed by low grade (G1) with 7/32 and two high-grade tumors (G3, both with SSR-overexpression). For three patients no grading was assessed. Further clinical information of patient cohort is given in Table 1. Both liver lobes were treated with TARE in 28 patients, and unilobar treatment was performed in four patients (three patients right lobe only and one patient left lobe only). TARE was performed for both liver lobes in two separate sessions (*n* = 26) or in one single session (*n* = 2). 

By study end, death was noted for 14 of the 32 (44%) patients, and hepatic progression on imaging was noted for 28 of the 32 (88%) patients. Overall median survival was 68.8 months (95% confidence interval (CI): 35.4 months, 102.2 months). One-year and two-year survival for the entire cohort was 100% and 84%, respectively. Median follow-up time was 58.7 months. Median HPFS was 21.5 months (95% CI: 9 months–34 months), median extrahepatic PFS was 13.1 m (95% CI: 11 months–15.2 months) and median overall PFS was 12.7 months (95% CI: 10.8 months–14.6 months). There were no significant differences in OS or HPFS between G1, G2, or G3 tumors. However, there was only a small number of patients with G3 tumors (2/32); these tended to have shorter OS and HPFS (Table 2).

### 3.2. Pre- and Postinterventional Measurements 

There were no significant changes of ADC values or SUV in tumor-free spleen and liver parenchyma, while intra-tumoral ADC increased and intra-tumoral SUV decreased after treatment (Table 3). Inter-reader agreement of ADC values was assessed by ICCs. Reliability between both readers was excellent (ADCmin ICC: 0.94, CI 0.92–0.96, ADCmean ICC: 0.89, CI 0.85–0.92). Percentage changes in ADCmin and SUVmean were weakly to moderately correlated (r = −0.38, *p* = 0.03), while the other parameters showed no significant correlation.

### 3.3. Response Assessment on First Follow-Up

**Figure 2:**
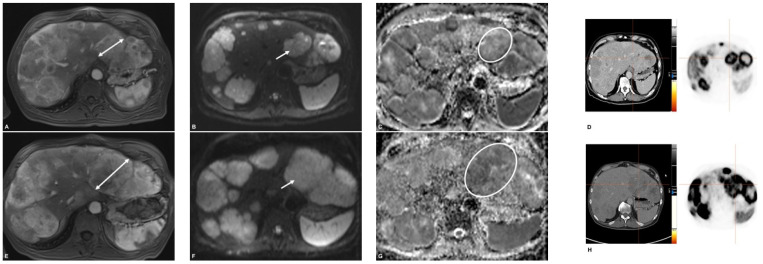
Patient with progressive disease. On arterial phase, lesion in the left lobe showed strong arterial enhancement (**A**) with diffusion restriction on diffusion-weighted image with b = 800 mm²/s (**B**) and low signal on ADC map (**C**). On PET/CT the lesion showed an SSR-overexpression (**D**). After TARE, there was a significant increase in size of this lesion with remaining arterial enhancement (**E**), restricted diffusion (**F**), low ADC signal (**G**), and increasing SSR overexpression (**H**). This lesion was rated as progressive disease.

**Figure 3:**
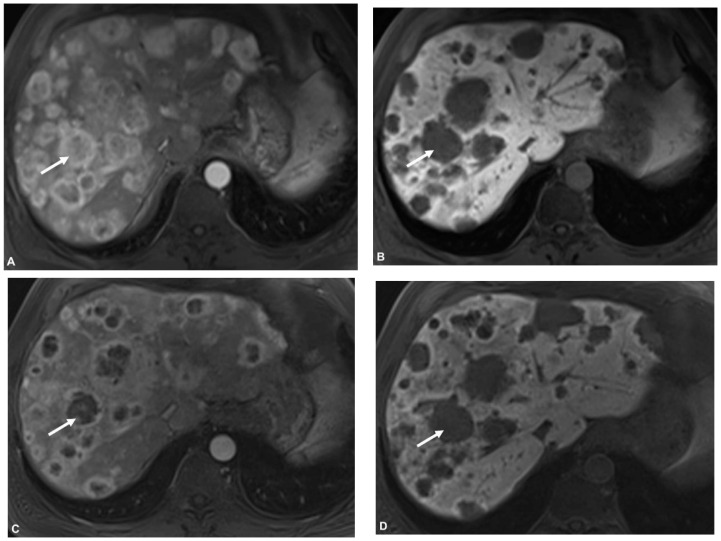
Patient with SD by RECIST vs. PR by mRECIST. On T1 weighted imaging on liver-specific phase the lesion showed low signal before (**B**) and after (**D**) TARE with not much change in size. However, there was an obvious decrease in arterial enhancement of the lesion pre- (**A**) and postinterventionally (**C**); therefore, the lesion was rated stable according to RECIST 1.1 criteria and as partial response according to mRECIST criteria.

**Figure 4:**
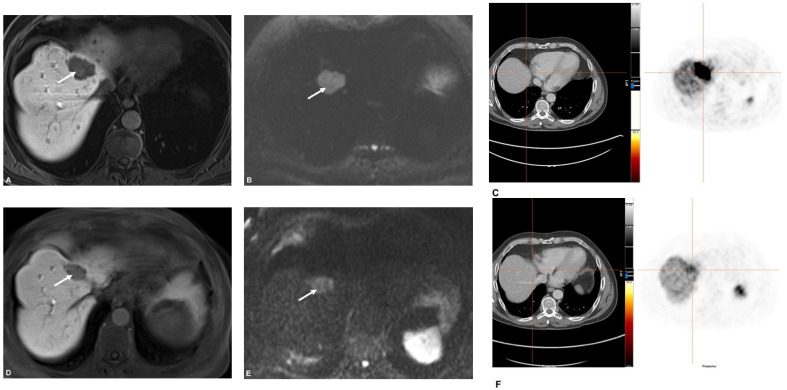
Patient with PR after TARE. In this patient, the liver metastasis showed hypointense signal on liver-specific phase (**A**), restricted diffusion (**B**), and SSR overexpression (**C**) before TARE. Postinterventionally, there was a significant shrinkage in size (**D**), less diffusion restriction (**E**), and a decrease of SSR overexpression (**F**) indicating partial response.

RECIST 1.1: The number of patients classified as responders (PR + SD) was 29 (91%) and 3 (9%) were classified as non- responders (PD) (Table 4). Responders tended to have a stronger increase of ADCmin values after TARE (23%; IQR −5–44%) compared to non-responders (−11%; IQR −13–15%), however statistical comparison was omitted due to inhomogeneous group sizes. In addition, responders showed a stronger decrease of SUVmax and SUVmean (ΔSUVmean −18%; IQR −36–1%) after treatment than non-responders ((ΔSUVmean 1%; IQR 1–41%) (Figure 2, Figure 3 and Figure 4). Percentage changes of tumor diameter according to RECIST showed a moderate positive correlation with percentage changes of SUVmean (r = 0.46, *p* = 0.009); percentage changes of ADCmean were weakly negatively correlated (r = −0.31, *p* = 0.09). 

mRECIST: Preinterventionally all analyzed lesions showed arterial enhancement. The number of patients classified as responders (PR + SD) was 26 (84%) and 5 (16%) were classified as non-responders (PD). ADC values (ADCmin and ADC mean) increased significantly in responders (*p* < 0.003), while there was no significant change of ADC in non-responders. Responders also had a stronger percentage decrease of SUV; for example, ΔSUVmean was −20% (IQR −39–0%) in responders compared to 1% (IQR −4–24%) in non-responders. 

**Figure 5:**
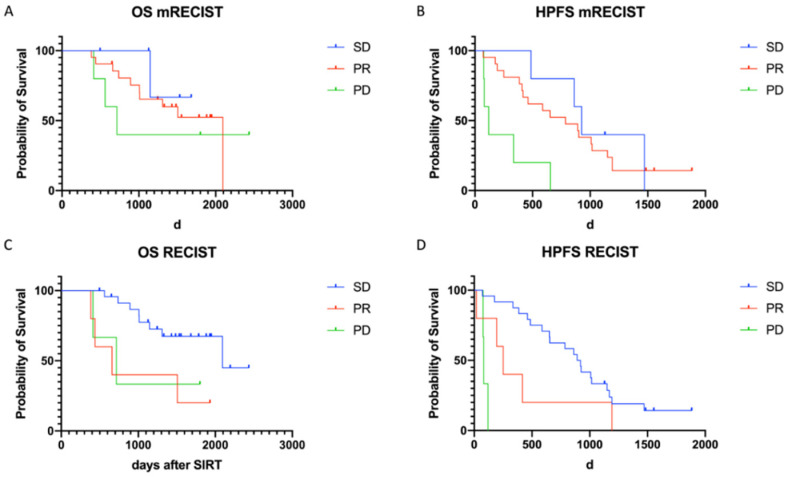
Kaplan–Meier curves of OS and HPFS of response groups by RECIST 1.1 (**C**,**D**) and mRECIST (**A**,**B**). According to RECIST, 1.1 patients classified as PR tended to have shorter HPFS than patients classified as SD (251 d vs. 906 d, *p* = 0.07) (**D**), while according to mRECIST, patients classified as PR or SD had similar HPFS (PR =786 d, SD = 926), which was significantly longer than that of patients classified as PD (119 d) (**B**).When evaluated by RECIST 1.1 patients classified as SD had longer OS than patients with PR (1879 d (95% CI: 1579–2180 d) vs. 984 d (95% CI: 439–1529 d)) (**C**). By contrast when evaluated by mRECIST OS between response groups did not differ significantly (PR: 1537d, SD: 1504d, PD: 1313 d).

However, neither classification according to RECIST 1.1. nor to mRECIST correlated with OS (Figure 5).

### 3.4. Response According to HPFS > 6 Months 

Of the 32 patients, 27 had an HPFS > 6 months (m) and were defined as responders. These patients did not show a significantly longer OS (*p* > 0.6). Responders showed a significant increase of ADCmean and ADCmin values (*p* < 0.003) in first follow-up after TARE, while there was no significant change of ADC values in non-responders (Table S1). SUVmax decreased significantly (*p* < 0.007) in responders, while there was no significant change in non-responders. SUV, tumor-to-organ ratios, and ADC were analyzed with ROC curves to obtain an optimal threshold to differentiate responders (HPFS > 6 m) from non-responders (Table 5). ΔT/L ratios (mean/mean) were found the best metrics, followed by ΔSUVmean and ΔADCmin. 

### 3.5. Response According to HPFS > 12 Months 

**Figure 6:**
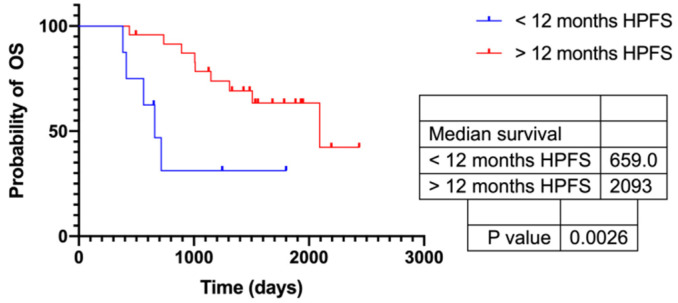
Kaplan–Meier curves of responder with an HPFS > 12 months and OS.

24 of the 32 patients were responders with an HPFS > 12 months and showed a significantly longer OS (Figure 6). ADCmean and ADCmin increased significantly in responders (*p* < 0.009), while there was no significant change in non-responders (Table S1). In addition, SUVmax and SUVmean decreased significantly after TARE in responders (*p* < 0.01), while there was no significant change in non-responders. Using ROC analysis, ΔT/L ratios (max/mean) and ΔT/L ratios (mean/mean) were found the best metrics (Table 6).

### 3.6. Response According to HPFS > Median (720 d)

**Figure 7:**
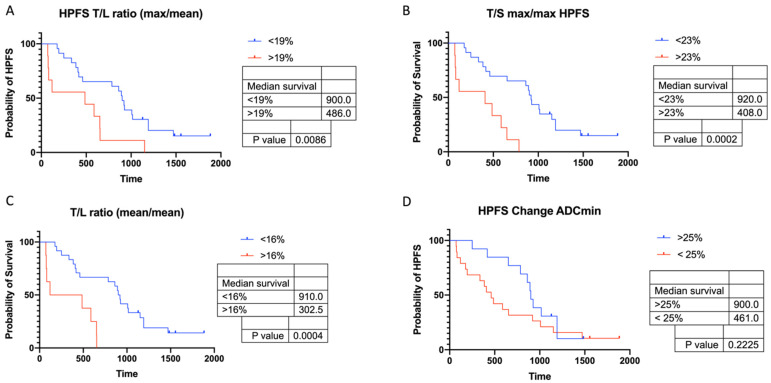
Kaplan–Meier curves for HPFS stratified by percentage changes of SUV and ADC between baseline and first follow-up after TARE. A lower percentage increase of SUV T/L ratio (max/mean), SUV T/L ratio (mean/mean), and SUV T/S ratio (max/max) showed significant effects on HPFS. (**A–C**) Patients with ΔT/S (max/max) > 23% had significantly shorter HPFS than those with ΔT/S (max/max) < 23% (**B**). Patients with an increase of ADCmin > 25% tended to have longer HPFS, although not significantly (**D**). *p* values were calculated with log-rank test.

16 of the 32 patients had an HPFS > 720 d and showed a significantly longer OS (*p* = 0.007). These 16 responders showed a significant increase of ADCmin and ADCmean (*p* = 0.02) and a slightly significant decrease of SUVmax and SUVmean (*p* < 0.04), while there were no significant changes in non-responders. Using ROC curves, ΔT/S ratios were found the best metrics (including ΔT/S ratio (max/mean), ΔT/S ratio (max/max), and ΔT/S ratio (mean/mean)) with an AUC of 0.7 followed by ΔT/L ratio (max/mean) and ΔADC (ADCmin and ADC mean) to discriminate patients with an above-median HPFS (Table 7). Patients with a ΔT/S ratio (max/max) < 23%, or a ΔT/L ratio (max/mean) < 19% had significantly longer HPFS (Figure 7). 

## 4. Discussion

Early evaluation of treatment response in NELM after TARE is especially challenging. Firstly, NET are slow-growing tumors and secondly, changes in tumor tissue after loco-regional treatments often show distinct response patterns compared to cytotoxic therapies. In addition, the value of laboratory markers such as chromogranin A to assess response is only moderate [10,25]. Therefore, evaluation of alternative imaging markers that might correlate better with PFS and OS is essential. Although the use of PET/CT with ^68^Ga-SSA is well-standardized for staging of NET, its use to predict therapy response remains indeterminate. Quantitative evaluation of ^68^Ga-DOTA-TATE PET/CT is not standardized with SUVmax or with tumor-to-spleen and tumor-to-liver ratios being reported in current research [9,10]. 

Our results showed that independently of the standard of reference for response (HPFS > 6 m, > 12 m, > 24 m) there was already a significant increase of ADC values and a significant decrease of SUVmax in responders in early response assessment (around 3 months after TARE) while there were no significant changes between pre- and postinterventional values in non-responders. Using ROC analysis, percentage changes of SUV tumor-to-organ ratios were found to be the best metrics to predict longer HPFS compared to ΔSUVmax/SUVmean alone and ΔADC. In particular, ΔT/L ratios (max/mean), and ΔT/S ratios (max/max) showed good AUC and had the most robust cut-off values (19% vs. 23%) over the three different time points analyzed in this study. Patients with a ΔT/S ratio (max/max) < 23% had a median HPFS of 920 d compared to 408 d with a ΔT/S ratio (max/max) > 23%. However, percentage changes of ADCmin were only slightly inferior as a diagnostic test, e.g., AUC of ΔADCmin for HPFS > 6 m was 0.79. Overall performance of ΔADCmin and ΔADCmean was similar. 

To our knowledge there is only one study by Filippi et al. that assessed the role of ^68^Ga-SSA PET/CT for response assessment in NELM after TARE. However, in contrast to our work, the authors analyzed ^68^Ga-DOTA-NOC instead of ^68^Ga-DOTA-TATE at baseline and 6 weeks after ^90^Y-RE and measured ΔT/S ratio (max/mean) as reference. They defined a molecular response as a reduction of >50% in ΔT/S ratio with responders showing a significantly longer OS and PFS [10]. However, in our study percentage decrease of ΔT/S ratio (max/mean) was not as high, and an optimal threshold according to our data would rather be >25% to define patients with longer HPFS. So, despite use of tumor-to-organ ratios, which are thought to be more scanner-independent, we could not reproduce the same thresholds for the ΔT/S ratio. However, these differences might be explained by different time points, different tracer accumulation, and a smaller study size and shorter observation period in their study. 

Other studies analyzed the value of ^68^Ga-SSA PET/CT for treatment evaluation following PRRT and treatment with octreotide. Haug et al. evaluated ^68^Ga-DOTA-TATE PET/CT for early response prediction after PRRT and also found ΔT/S ratios (max/max) to be superior to ΔSUVmax to predict patient outcome. Patients with a decline in ΔT/S ratio had a significantly longer PFS than patients with stable or increased ΔSUV T/S ratios [9]. 

In our study overall median survival was rather long at 69 months (95% CI: 35.4 months, 102.2 months). A recent meta-analysis, including 21 studies with NELM following TARE, reported a median OS of 29.2 m (range 12.5–70 m) [26]. Overall median PFS rate in our study was 12.7 m which was in line with recently reported rates of 11.3 m [27]. However, we found no studies which separately analyzed hepatic and extrahepatic PFS in NELM after TARE, which in our opinion is interesting after a locoregional (vs. systemic) treatment strategy. 

As also reported by Braat et al. we detected higher rates of patients classified as PR on first follow-up when evaluated according to mRECIST compared to RECIST 1.1 (Figure 3), however differently to some reports we had no patients rated as CR after TARE. In our study radioembolization resulted in PR in 16%, SD in 75%, and PD in 9% based on RECIST 1.1, and in PR in 68%, SD in 16%, and PD 16% according to mRECIST. These rates were comparable to those reported by Braat et al. in 244 patients with CR in 2%, PR in 14%, SD in 75%, and PD in 9% according to RECIST and CR in 8%, PR in 35%, SD in 48%, and PD in 9% according to mRECIST [28]. 

The definition of responder vs. non-responder by RECIST is applied quite incongruent among studies. The commonly used endpoint is objective response rate (ORR) which is defined as CR + PR. A recent meta-analysis by Pollock et al. identified ORR as a predictor for OS in patients with NELM after TARE with Yttrium-^90^ resin microspheres [26]. However, in our study neither classification by mRECIST nor RECIST1.1. had a prognostic value regarding OS. When evaluated by RECIST patients classified as SD showed even longer OS and HPFS than patients classified as PR (Figure 5). Also, in a non-curative setting it is debatable to define stable disease as non-responders, therefore we defined responders in this study as PR + SD.

A recent study by Huizing et al. evaluating response assessment in NET after PRRT found similar results when assessing treatment response by RECIST 1.1. after 3 m, while progression evaluated after 9 m was associated with worse OS [29]. Gowdra Halappa et al. [19] also found no differences in survival in GEP-NETs after TARE according to mRECIST. In addition, classification by RECIST 1.1 and mRECIST did not show a good correlation with HPFS. Of course, HPFS of patients defined as PD was significantly shorter according to both classification systems, as we analyzed radiological hepatic progression according to RECIST. However, HPFS of PR was longer when evaluated according to mRECIST (786 d) compared to RECIST (251 d), which might indicate that response assessment according to mRECIST is superior. However, these results underline the need for better strategies in assessing treatment response. 

Imaoka et al. showed, in two meta-analyses, that there was a strong correlation between 12-months PFS and median PFS, while there was no significant relationship between objective response rate (ORR) according to RECIST and median PFS. Also, PFS and OS correlated significantly, while ORR showed no correlation with OS; thus the authors concluded that PFS is a good surrogate for OS and 12-months PFS rates represent acceptable alternate endpoints for clinical trials [27,30]. Our study confirms these suggestions, as patients with a HPFS > 12 months/24 months had a significantly longer OS and patients with an HPFS > 6 months had a significantly longer median HPFS. 

The difficulty we encounter in NETs, is to define an ideal standard of reference; OS might be limited since we have comparatively long OS and different post-salvage treatments influence this parameter; HPFS on the other hand is size based—however detection of new lesions is independent of size. 

Due to the retrospective design of our study, time-intervals between pre- and interventional examinations and between MRI and PET/CT are not homogeneous and not all patients were examined on the same scanners. Also, pre- and postinterventional therapies were slightly different, although treatment decisions were made in a multidisciplinary and certified tumor board. Altogether, the results should not be significantly influenced by this—underlined by a good significance level—and, on the other hand, it reflects the clinical routine. Another limitation is that patients undergoing TARE were only acquired until September 2017 for this study, however this allowed us to analyze rather long OS and PFS data and general imaging techniques have not changed in recent years. However, prospective trials with larger cohort and a multicenter setting are needed to confirm our results. In addition, it would be interesting to evaluate the prognostic value of preinterventional PET/CT and MRI parameters regarding their value for outcome prediction in further studies in a larger patient cohort. There are already promising studies which could demonstrate that ADC values allowed survival prognosis in colorectal liver metastases treated with ^90^Y-micosphere radioembolization [31] as well as that ADC could be used as predictor for response to chemotherapy of liver metastases in colorectal cancer [32].

Our results show that ^68^Ga DOTATATE PET/CT and MRI including DWI allow early and robust evaluation of tumor response whereby the prognostic value by using SUV ratios of T/S seems to be the best imaging marker. However, PET/CT is limited in terms of availability, radiation exposure, and cost efficiency compared to MRI. In addition, reduced uptake of tracer in the tumor may lead to a higher uptake in unaffected spleen or other organs which may affect SUV calculations [9]. Furthermore, not all metastatic NET show SSR overexpression, especially if they are high-graded or part of a dedifferentiation under therapy. It was found that the ratio measurements (especially the ratio T/S) were superior to the absolute measurements (tumor SUVmax or SUVmean). In patients with splenectomy, T/L ratio (max/mean) represents a good alternative. 

On the other hand, DWI is highly prone to artifacts and ADC measurements and their reproducibility can vary, for example, due to different scan parameters. However, several previous studies showed only minor differences of ADC values, even between different MRI scanners [33]. 

## 5. Conclusions

Our results indicate that DWI and ^68^Ga DOTATATE PET/CT may be feasible for short- and long-term assessment of therapy response after TARE in patients with NET. 

## Figures and Tables

**Table 1:** Clinical features of patients before TARE.

Clinical Features	Classification	Number of Patients
Liver intervention	None	27
	RFA	1
	TARE	4
	TACE	1
Systemic therapy	None	4
	PRRT	12
	Biotherapy	16
	Chemotherapy	6
Extrahepatic metastases	None	7
	Lymph nodes	15
	Peritoneal	5
	Bone	12
	Pulmonal	1
	Mesenterial	5
	Other	2
Intrahepatic tumor load	<10%	7
	10–25%	13
	25–50%	9
	51–75%	3
	>75%	0

**Table 2:** OS and HPFS according to histopathological grading of patients.

Tumor Grade	Number	OS	HPFS
G1	7	57.7 months (95% CI: 42.6–72.7 months)	29.5 months (95% CI: 18–41 months)
G2	20	50.7 months (95% CI: 40.2–61.3 months)	26 months (95% CI: 18.3–33.8 months)
G3	2	23.5 months (95% CI: 23.5–23.5 months)	9.3 months (95% CI: 0.0–22.4 months)

**Table 3:** Pre- and postinterventional imaging parameters.

Imaging Parameters	Pretreatment	Posttreatment	Change (%)	*p*-Value
Size (mm) ^1^	32.1(±12.4)	28.8 (±11)	−6 (−22–−2)	0.009
Tumor ADCmin ^2^	0.74 (±0.24)	0.89 (±0.29)	18.3 (−7.6–40.4)	0.003
Tumor ADCmean ^2^	0.88 (±0.29)	1.05 (±0.31)	14.2 (−2–44.6)	0.003
Liver ADCmean ^2^	0.97 (±0.22)	0.99 (±0.20)		0.602
Tumor SUVmax	29.4 (±16.8)	23.2 (±13.3)	−22.5 (−35.8–1.4)	0.005
Tumor SUVmean	16.2 (±8.4)	13.1 (±6.5)	−14.7 (−34.2–2.2)	0.009
Liver SUVmax	7.4 (±2.4)	7.2 (±3.1)		0.700
Liver SUVmean	5.7 (±1.6)	5.5 (±2.1)		0.767
Spleen SUVmax	21.9 (±10.5)	19.9 (±9.0)		0.176
Spleen SUVmean	17.3 (±8.4)	17.5 (18.0)		0.952
T/L (max/max)	4.2 (±2.7)	3.8 (±3.0)	−18.2 (−41.7–22.5)	0.566
T/S (max/max)	1.7 (±1.3)	1.6(±1.3)	−3.0 (−43.9–37.8)	0.488
T/L (max/mean)	5.5 (±3.3)	4.6 (±3.1)	−18.4 (−44.5–21.9)	0.080
T/S (max/mean)	2.2 (± 1.8)	2.0 (± 1.6)	−13.1 (−37.7–32.1)	0.355
T/L (mean/mean)	3.0 (± 1.6)	2.6 (±1.6)	−17.6 (−38.8–18.7)	0.048
T/S (mean/mean)	1.2 (±0.9)	1.2 (±0.9)	−4.4 (−36.1–29.6)	0.519

^1^ Averaged for three target lesions per patient; ^2^ mean values of ADCmin and ADCmean are given in 10^−3^ mm^2^/s.

**Table 4:** Distribution of treatment response on first follow-up by RECIST 1.1 and mRECIST.

Treatment Response	RECIST 1.1	mRECIST
PR	5	21
SD	24	5
PD	3	5
Not analyzed	0	1 ^1^

^1^ Injection of contrast medium not possible.

**Table 5:** ROC analysis of SUV and ADC for predicting HPFS < 6 months.

	ΔSUVmean	ΔT/L Ratio (Mean/Mean)	ΔT/L Ratio (Max/Mean)	ΔT/S Ratio (Max/Max)	ΔADCmin	ΔADCmean
Best cut-off (%)	>−8	>24	>19	>23	<16	<18
Sensitivity (%)	100	80	80	80	100	100
Specificity (%)	70	93	82	82	63	56
AUC	0.79	0.82	0.76	0.72	0.79	0.70

**Table 6:** ROC analysis of SUV and ADC for predicting HPFS < 12 months.

	ΔSUVmean	ΔT/L Ratio (Mean/Mean)	ΔT/L Ratio (Max/Mean)	ΔT/S Ratio (Max/Max)	ΔADCmin	ΔADCmean
Best cut-off (%)	>−8	>24	>19	>23	<22	<20
Sensitivity (%)	63	50	50	50	75	88
Specificity (%)	67	92	79	79	54	54
AUC	0.56	0.64	0.65	0.61	0.60	0.59

**Table 7:** ROC analysis of SUV and ADC for predicting HPFS < 24 months.

	ΔSUVmean	ΔT/L Ratio (Mean/Mean)	ΔT/L Ratio (Max/Mean)	ΔT/S Ratio (Max/Max)	ΔADCmin	ΔADCmean
Best cut-off (%)	>−8	>16	>19	>23	<22	<25
Sensitivity (%)	56	50	50	50	75	81
Specificity (%)	75	100	94	94	69	63
AUC	0.57	0.63	0.64	0.7	0.66	0.63

## Data Availability

The data presented in this study are available on request from the corresponding author.

## Supplementary Materials

The following are available online at https://www.mdpi.com/article/10.3390/cancers13174321/s1, Table S1: Pre- and postinterventional ADC characteristics and response groups.
