# Supplementary material for: 68Ga-DOTATATE PET/CT and MRI with Diffusion-Weighted Imaging (DWI) in Short- and Long-Term Assessment of Tumor Response of Neuroendocrine Liver Metastases (NELM) Following Transarterial Radioembolization (TARE)
Source: Cancers (Basel). 2021 Aug 27;13(17):4321. doi: 10.3390/cancers13174321 (PMC8431353; doi:10.3390/cancers13174321)
Supplement: Supplementary file 1 [file cancers-13-04321-s001.zip › cancers-1329396-supplementary.pdf]

Supplementary Material: ^{68}Ga-DOTATATE PET/CT and MRI with Diffusion-Weighted Imaging (DWI) in Short- and Long-Term Assessment of Tumor Response of Neuroendocrine Liver Metastases (NELM) Following Transarterial Radioembolization (TARE)

Maria Ingenerf, Sophia Kiesl, Salma Karim, Leonie Beyer, Harun Ilhan, Johannes Rübenthaler, Max Seidensticker, Jens Ricke and Christine Schmid-Tannwald

Table S1. Pre- and postinterventional ADC characteristics and response groups.

	RECIST 1.1.	mRECIST	ADCmean Pre	ADC Mean Post	% ADC Mean	ADCmin Pre	ADCmin Post	% ADC Min
1	SD	n.a.	0.81	0.67	−17.08	0.69	0.61	−11.53
2	SD	SD	0.50	1.18	138.24	0.32	0.90	181.29
3	SD	PR	1.09	1.16	6.74	0.75	0.72	−3.88
4	SD	PR	0.70	1.67	140.91	0.51	1.43	178.93
5	SD	PR	0.61	0.83	35.52	0.55	0.69	25.78
6	SD	PR	0.92	0.98	6.56	0.82	0.76	−7.92
7	SD	PR	0.52	0.86	66.31	0.52	0.77	46.98
8	SD	SD	1.12	1.73	54.80	1.10	1.61	46.65
9	SD	SD	0.63	0.63	0.26	0.55	0.56	1.72
10	SD	PD	0.67	0.78	16.51	0.53	0.71	34.02
11	SD	PR	0.58	0.56	−2.25	0.45	0.48	6.10
12	PR	PR	0.76	1.18	54.50	0.60	0.98	63.83
13	SD	SD	0.55	0.73	32.80	0.50	0.67	33.73
14	PR	PR	1.00	1.11	11.21	0.86	1.04	20.93
15	SD	PR	0.59	0.78	34.09	0.49	0.68	39.99
16	SD	PR	1.81	1.43	−20.64	1.35	1.26	−6.57
17	SD	PR	1.17	1.17	−0.48	0.87	1.07	22.92
18	SD	SD	1.49	1.29	−13.07	1.24	1.07	−13.90
19	SD	PR	0.92	0.86	−6.29	0.73	0.64	−11.85
20	SD	PR	0.94	0.73	−22.74	0.79	0.62	−21.29
21	PR	PR	1.08	1.61	48.41	0.70	1.48	113.37
22	SD	PR	0.93	1.19	28.02	0.84	1.09	28.53
23	PR	PR	0.65	0.96	46.89	0.57	0.80	40.53
24	SD	PR	0.86	1.18	37.74	0.74	1.04	39.86
25	SD	PR	0.49	0.60	21.74	0.46	0.33	−28.88
26	SD	PR	1.18	1.33	12.35	1.05	1.03	−1.80
27	SD	PD	0.87	0.92	6.28	0.76	0.73	−3.44
28	SD	PR	0.94	0.99	5.37	0.75	0.86	15.59
29	PR	PR	0.79	1.23	55.98	0.65	1.00	54.10
30	PD	PD	1.14	1.32	16.12	1.05	1.20	14.53
31	PD	PD	1.07	1.04	−2.92	0.99	0.86	−13.22
32	PD	PD	0.87	0.75	−13.13	0.80	0.71	−10.81
